# Mirabolides A and B; New Cytotoxic Glycerides from the Red Sea Sponge *Theonella mirabilis*

**DOI:** 10.3390/md14080155

**Published:** 2016-08-18

**Authors:** Dina R. Abou-Hussein, Diaa T. A. Youssef

**Affiliations:** 1 Department of Natural Products and Alternative Medicine, Faculty of Pharmacy, King Abdulaziz University, Jeddah 21589, Saudi Arabia; dabouhussein@kau.edu.sa or dina.abouhussein@pharma.cu.edu.eg; 2 Department of Pharmacognosy, Faculty of Pharmacy, Cairo University, Kasr El-Aini, Cairo 11562, Egypt

**Keywords:** Red Sea sponge *Theonella mirabilis*, glycerides, Mirabolides A and B, 4-methylene sterols, breast cancer cell line, cytotoxic activity

## Abstract

As a part of our continuing work to find out bioactive lead molecules from marine invertebrates, the CHCl_3_ fraction of the organic extract of the Red Sea sponge *Theonella mirabilis* showed cytotoxic activity in our primary screen. Bioassay-guided purification of the active fractions of the sponge’s extract resulted in the isolation of two new glycerides, mirabolides A and B (**1** and **2**), together with the reported 4-methylene sterols, conicasterol (**3**) and swinhosterol B (**4**). The structures of the compounds were assigned by interpretation of their 1D (^1^H, ^13^C), 2D (COSY, HSQC, HMBC, ROESY) NMR spectral data and high-resolution mass determinations. Compounds **1**–**4** displayed marked cytotoxic activity against human breast adenocarcinoma cell line (MCF-7) with IC_50_ values of 16.4, 5.18, 6.23 and 3.0 μg/mL, respectively, compared to 5.4 μg/mL observed by doxorubicin as reference drug.

## 1. Introduction

Marine sponges are multicellular animals evolved from about 600 million years ago; they belong to phylum Porifera and are considered the most primitive animals [1]. More than 8000 species of sponges have been described and are classified into three classes [2]. Diverse classes of bioactive metabolites were isolated from sponges and reported in the literature in the thousands; they proved that sponges have been essential and important source of drug discovery [3].

Sponges of order Lithistida are characterized by a hard rocky skeleton formed by the interlocking spicules of silica (desmas) [4,5]. They occur worldwide and are recognized for their different bioactive metabolites such as polyketides, peptides (linear or cyclic), macrolides, alkaloids, lipids, steroids and pigments [4,6,7,8]. Genus *Theonella* is well-known producer of diverse bioactive constituents including 4-methylene sterols [9], cyclic or linear peptides [10], and fatty acids [11,12].

Several metabolites isolated from the genus *Theonella* demonstrated several cytotoxic activities *viz*. macrolides [13], cyclopeptides [14,15,16], cyclodepsipeptides [17,18], linear peptides [19], dimeric macrolides [20], and alkaloids [21]. Certain depsipetides were proven to protect cells from HIV infection [18]; others exhibited antifungal and antibacterial activities [22]. Glycolipids with five-membered cyclitol were previously isolated from the Carribean sponge *Pseudocertina crassa* and were known as crasserides [23]. Crasserides and isocrasserides were verified to be present in different investigated species of marine sponges and then were distinctive to the phylum Porifera [24]. They can be considered as natural feeding deterrents due of their exerted antifeedant activity on the fish *Carassius auratus* [23].

In the course of our ongoing quest to discover cytotoxic compounds from Red Sea marine lithistid sponges [25,26], the crude extract of the sponge *Theonella mirabilis* showed cytotoxic activity with IC_50_ of 17 μg/mL against human breast cancer cell line (MCF-7). Bioassay-directed fractionation of the active fractions afforded four compounds including two new glycerides, mirabolides A and B (**1** and **2**) and the known conicasterol (**3**) [27] and swinhosterol B (**4**) [28]. Herein, we report on the purification, structure determination as well as the cytotoxic activities of these compounds.

## 2. Results and Discussion

**Figure 1:**
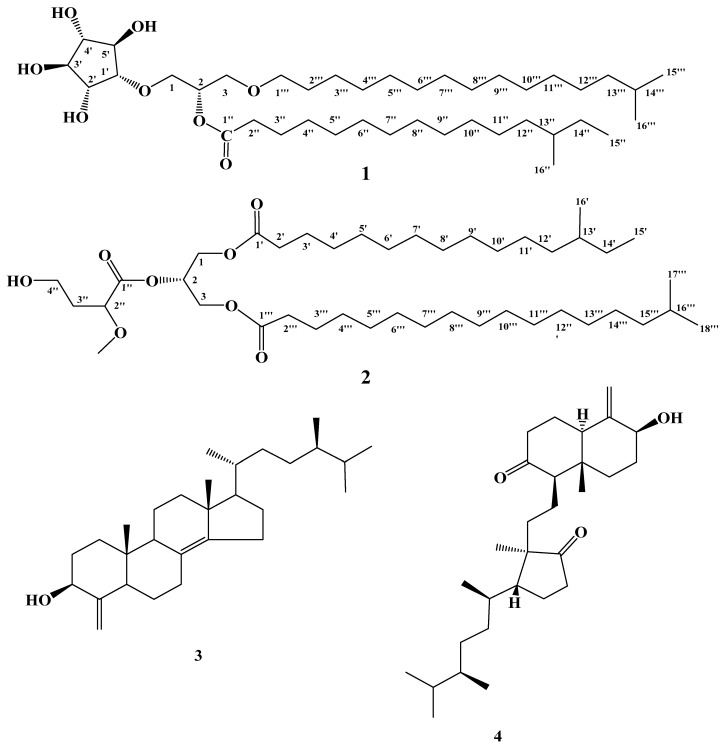
Structures of compounds **1**–**4**.

**Figure 2:**
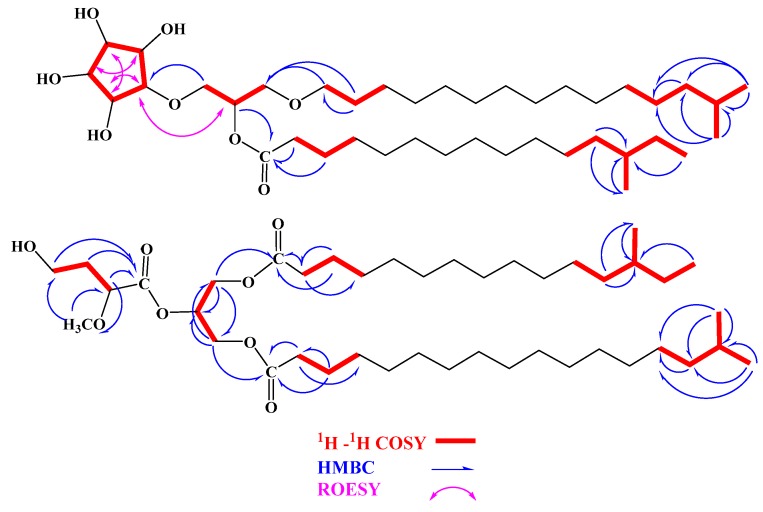
Key COSY, HMBC and ROESY correlations of **1** and **2**.

Compound **1** (Figure 1) was obtained as a white precipitate. Its molecular formula was assigned as C_40_H_78_O_8_ as deduced by HRESIMS pseudomolecular ion peak at *m/z* 685.5625 [M − H]^−^, suggesting two degrees of unsaturation. Combined 1D (^1^H and ^13^C) and 2D (COSY, HSQC, HMBC, ROESY) NMR data allowed the assignment of a trisubstituted glycerol moiety attached to three subunits including, a five-membered cyclitol moiety, an *O*-acyl chain (13-methylpentadecanoyl) and an *O*-alkyl chain (14-methylpentadecanyl) (Figure 1 and Table 1). The signals at δ_H_/δ_C_ 3.78,3.73/67.7 (H_2_-1/C-1), 5.17/69.3 (H-2/C-2) and 3.57,3.54/67.1 (H_2_-3/C-3) were assigned as a trisubstituted glycerol moiety (Supplementary Materials, Figures S1 and S2) [23,24]. The signals at δ_H_/δ_C_ 3.66/80.3 (H-1′/C-1′), 3.93/71.4 (H-2′/C-2′), 3.84/78.5 (H-3′/C-3′), 3.69/77.0 (H-4′/C-4′) and 3.90/77.2 (H-5′/C-5′) were assigned as cyclopentane-1,2,3,4,5-pentaol [23,24]. The assignment of these signals was secured from COSY, and HSQC experiment (Supplementary Materials, Figures S3 and S4). The COSY spectrum showed non-interrupted ^1^H–^1^H correlations from H-1′ to H-5′ through H-2′, H-3′ and H-4′ suggesting a five-membered cyclitol moiety [23,24]. The placement of this moiety at C-1 was unambiguously secured from HMBC correlation of H_2_-1/C-1′ (Figure 2). The configuration of the cyclitol in **1** moiety was assigned from ROESY experiment as well as by comparison of the ^1^H/^13^C NMR data as well as the coupling constants of H-1′→H-5′ with those in the literature [23,24]. The ROESY correlations between H-1′ and H-2′, H-2′ and H-4′ as well as between H-3′ and H-5′ (Figure 2, Supplementary Materials, Figure S9) supported the configuration at the cyclitol moiety which was in agreement with the reported one in crasserides [23]. The fatty acyl moiety at C-2 was assigned as 13-methylpentadecanoyl based upon the signals at δ_H_/δ_C_ 172.1 (C-1″), 2.33/32.5 (H_2_-2″/C-2″), 1.60/23.0 (H_2_-3″/C-3″), 1.25/20.8–35.1 (H_2_-4″-H_2_-11″/C-4″-C-11″), 1.06/35.2 (H_2_-12″/C-12″), 1.35/30.8 (H-13″/C-13″), 1.25/30.0 (H_2_-14″/C-14″), 0.88/12.2 (H_3_-15″/C-15″) and 0.83/17.7 (H_3_-16″/C-16″). Further COSY correlations and HMBC cross peaks (Figure 2) of H_2_-2″/C-1″ and H_2_-3″/C-1″ supported this assignment. In addition, HMBC cross peaks of H_2_-12″/C-13″, H_3_-15″/C-13″, H_3_-16″/C-13″ and H_2_-12″/C-16″ together with COSY correlations indicated the presence of an isobutyl moiety at the end of the acyl chain. The linkage of the ester moiety at C-2 was secured from HMBC cross peak of H-2/C-1″ (Figure 2, Supplementary Materials, Figures S5–S8).

Similarly, the assignment of the alkyl moiety at C-3 was established by 1D and 2D NMR and was identified as 14-methylpentadecanyl (Table 1 and Figure 2). The ^1^H and ^13^C NMR signals resonating at δ_H_/δ_C_ 3.45, 3.41/70.0 (H_2_-1′′′/C-1′′′), 1.53/27.8 (H_2_-2′′′/C-2′′′), 1.15/37.1 (H_2_-13′′′/C-13′′′), 1.50/26.0 (H-14′′′/C-14′′′), 0.85/20.7 (H_3_-15′′′/C-15′′′), 0.85/20.7 (H_3_-16′′′/C-16′′′) together with the methylene cluster at 1.25/20.8–35.1 (H_2_-3′′′-H_2_-12′′′/C-3′′′-C-12′′′) supported this assignment. The two methyl doublet signals at δ 0.85 (H_3_-15′′′ and H_3_-16′′′) indicated the iso-branching of the fatty chain. This was verified by COSY correlations and HMBC cross peaks (Table 1 and Figure 2) of H_2_-2′′′/C-1′′′, H_2_-13′′′/C-12′′′, H_3_-15′′′/C-12′′′, H_3_-16′′′/C-12′′′, H_3_-15′′′/C-13′′′, H_3_-16′′′/C-13′′′, H_3_-15′′′/C-14′′′ and H_3_-16′′′/C-14′′′. Again, the placement of the alkyl moiety at C-3 was supported by HMBC cross peak of H_2_-3/C-1′′′ (Figure 2, Supplementary Materials, Figures S5–S8), completing the structure of **1**.

**Figure 3:**
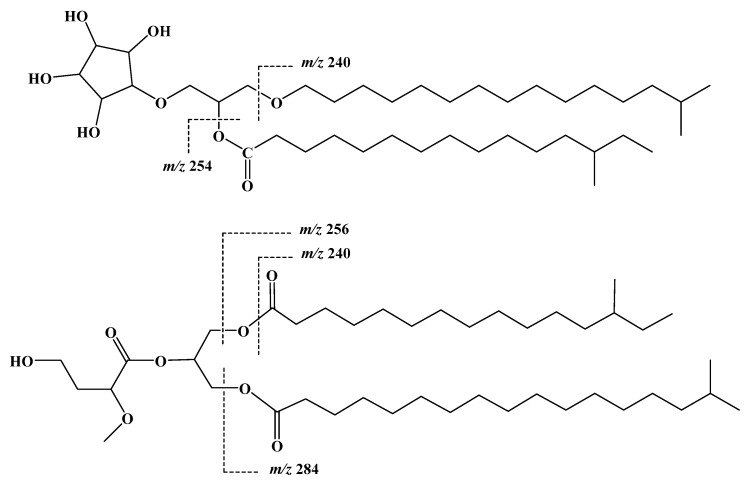
Key MS fragment ion peaks of **1** and **2**.

In the negative ESIMS of **1**, the presence of fragment ion peaks at *m*/*z* 254 and 240 supported the presence of alkyl side chains at C-2 and C-3, respectively (Figure 3). The overlapping of the signals of methylene cluster (4″–11″ and 3′′′–12′′′) of both alkyl chains at δ_H_/δ_C_ 1.25/20.8–35.1 and the lack of clear COSY correlations on both side chains in this region, necessitated carrying out methanolysis of **1** and methylation of the liberated fatty acids followed by GLC-MS analysis of the hydrolysate for an accurate determination of the length and the position of branching of the acyl chain. The obtained fatty methyl ester (methyl 13-methylpentadecanoate, *m*/*z* 270) was identified based on its GLC retention time and mass fragmentation spectrum. Two relatively intense fragment peaks at *m*/*z* 213 and 241 indicated the favored *α*-cleavage with the respect of the tertiary carbon atom (C-13″) carrying the methyl branch. Characteristic peaks at *m*/*z* 219 and 201 resulted from the loss of methanol and water from the fragment at *m*/*z* 241.

To the best of our knowledge, **1** is reported here as a new natural product and was named mirabolide A.

Compound **2** (Figure 1) was obtained as a white precipitate. It showed a molecular formula C_42_H_80_O_8_ as deduced from HRESIMS pseudomolecular ion peak at *m*/*z* 713.5933 [M + H]^+^, suggesting three degrees of unsaturation. Investigation of the 1D (^1^H and ^13^C) and 2D (COSY, HSQC and HMBC) NMR experiments allowed the assignment of a triglyceride skeleton (Table 2). The signals at δ_H_/δ_C_ 3.60/70.5 (H_2_-1/C-1), 5.22/71.5 (H-2/C-2) and 4.40, 4.16/63.9 (H_2_-3/C-3) were indicative for a trisubstituted glycerol moiety (Supplementary Materials, Figures S10 and S11) [23,24]. Extensive study of the 1D and 2D NMR allowed the assignment of three fatty acyl chains as 4-hydroxy-2-methoxybutanoyl, 13-methylpentadecanoyl and 16-methylheptadecanoyl. The acyl moiety at C-1 was assigned as 13-methylpentadecanoyl, based upon the signals δ_H_/δ_C_ at 175.1 (C-1′), 2.30/35.0 (H_2_-2′/C-2′), 1.59/26.1 (H_2_-3′/C-3′), 1.28/28.2–31.1 (H_2_-4′-H_2_-11′/C-4′-C-11′), 1.10/38.2 (H_2_-12′/C-12′), 1.31/35.7 (H-13′/C-13′), 1.28/30.8 (H_2_-14′/C-14′), 0.88/11.8 (H_3_-15′/C-15′) and 0.85/19.6 (H_3_-16′/C-16′). The COSY correlations and HMBC cross peaks of H-2′/C-1′, H_2_-3′/C-1′ supported this assignment (Figure 2). Moreover, the correlations in COSY, HSQC and HMBC experiments (Supplementary Materials, Figures S12–S17) of the two methyl groups at positions 13′ and 14′ indicated the presence of *sec*-butyl fragment at the end of the acyl chain. This was secured from HMBC cross peaks of H_2_-12′/C-13′, H_3_-15′/C-13′/, H_3_-16′/C-13′ and H_2_-12′/C-16′ (Table 2 and Figure 2). Finally, the placement of the fatty acid at C-1 was secured from HMBC correlation of H_2_-1/C-1′ (Table 2 and Figure 2). Similarly, the acyl moiety at C-3 was assigned as 16-methylheptadecanoyl. ^1^H and ^13^C NMR signals of this moiety showed resonating signals at δ_H_/δ_C_ 174.7 (C-1′′′), 2.30/35.1 (H_2_-2′′′/C-2′′′), 1.59/26.1 (H_2_-3′′′/C-3′′′), 1.28/28.2–31.1 (H_2_-4′′′-H_2_-14′′′/C-4′′′-C-14′′′), 1.19/40.2 (H_2_-15′′′/C-15′′′), 1.50/29.1 (H-16′′′/C-16′′′), 0.87/23.1 (H_3_-17′′′/C-17′′′) and 0.87/23.1 (H_3_-18′′′/C-18′′′). Two methyl doublets at δ 0.87 (H_3_-17′′′ and H_3_-18′′′) indicated the iso-branching of the fatty chain. The assignment was supported by COSY and HMBC cross peaks (Table 2 and Figure 2) of H-2′′′/C-1′′′and H_2_-3′′′/C-1′′′, in addition to the cross peaks of H_2_-15′′′/C-14′′′, H_3_-17′′′/C-14′′′, H_3_-17′′′/C-15′′′, H_3_-18′′′/C-15′′′, H_3_-17′′′/C-16′′′ and H_3_-18′′′/C-16′′′ (Table 2 and Figure 2). The placement of this moiety at C-3 was supported by HMBC of H_2_-3/C-1′′′ (Table 2 and Figure 2). Finally, the signals at δ_H_/δ_C_ 171.6 (C-1″), 3.70/77.5 (H-2″/C-2″), 2.08, 2.22/29.1 (H_2_-3″/C-3″), 3.53, 3.67/68.6 (H_2_-4″/C-4″) and 3.20/52.4 (H_3_CO-2″) were assigned as 4-hydroxy-2-methoxybutanoyl moiety attached to position 2 of the substituted glycerol. The assignment of this moiety was deduced from HMBC cross peaks of H-2″/C-1″, H_2_-3″/C-1″ and H_3_CO-2″/C-2″ (Figure 2, Supplementary Materials, Figures S14–S17). Evidence for the length of the two fatty acyl chains attached to C-1 and C-3 was provided by ESIMS fragmentation pattern of compound **2** and by GLC-MS analysis of the methyl esters produced after methanolysis. In the positive mode, fragment ion peaks at *m*/*z* 256 and 284 were evident for the presence of alkyl side chains at C-1 and C-3, respectively (Figure 3). GLC-MS analysis of the methylated fatty acids obtained after methanolysis and methylation of **2** was shown to comprise three methyl esters identified as methyl-2,4-dimethoxybutanoate (*m*/*z* 162), methyl-13-methyl-pentadecanoate (*m*/*z* 270) and methyl 16-methylheptadecanoate (*m*/*z* 298), corresponding to the three fatty acyl chains attached to C-2, C-1 and C-3, respectively. Branching of methyl at position C-13′ of methyl-13-methyl-pentadecanoate was indicated by the mass fragmentation pattern as in **1**.

To the best of our knowledge, **2** is reported here as a new natural product and was named mirabolide B.

The structures of compounds **3** and **4** (Figure 1) were assigned by interpretation of the 1D and 2D NMR data and MS as well as by comparison with literature data. Thus, compounds **3** and **4** were identified as conicasterol [27], and swinhosterol B [28], respectively.

Compounds **1**–**4** were evaluated for their cytotoxic activity against human breast adenocarcinoma cell line (MCF-7). Compound **4** was the most active compound with IC_50_ value of 3.0 μg/mL compared to doxorubicin (IC_50_ = 5.4 μg/mL). Compounds **1–3** were less active with IC_50_ values of 16.4, 5.18 and 6.23 μg/mL, respectively.

## 3. Materials and Methods

### 3.1. General Experimental Procedure

Optical rotations were recorded by a P800 series digital automatic high-speed polarimeter (A. KRÜSS Optronic, Hamburg, Germany). UV spectra were recorded on a Hitachi 300 spectrometer (Hitachi High-Technologies Corporation, Kyoto, Japan). IR spectral analysis were carried on Nicolet™ iS™10 FT-IR spectrometer (Thermo Scientific, Waltham, MA, USA); ESIMS spectra were obtained with a LCQ DECA mass spectrometer (ThermoFinnigan, Bremen, Germany) coupled to an Agilent 1100 HPLC system equipped with a photodiode array detector. Mass spectral data were obtained with a Micromass Q-tof equipped with lockspray mass spectrometer using Leucine Enkaphalin at *m*/*z* 556.2771 [M + H]^+^ as a reference mass. NMR spectra were obtained in CD_3_OD or CDCl_3_ on Bruker Avance DRX 600 MHz spectrometer (Bruker, Rheinstetten, Germany) at 600 MHz for ^1^H NMR and 150 MHz for ^13^C NMR. NMR spectra were referenced to the residual protonated solvent signals (CH_3_OH: 3.30 ppm for ^1^H and 49.0 ppm for ^13^C, CHCl_3_: 7.25 ppm for ^1^H and 77.23 ppm for ^13^C). Silica gel (Sigma-Aldrich, Darmstadt, Germany, 70–230 Mesh) and silica gel (Sigma-Aldrich, Darmstadt, Germany, 23–400 Mesh) were used for isolation of compounds on vacuum liquid chromatography and column chromatography, respectively. The HPLC separation was performed on a RP18, 250 × 10 mm, 5 μm Cosmosil ARII column (Nacalai Inc., San Diego, CA, USA). Trace Ultra GC system (Thermo Scientific, Surrey, UK) was used for GC analysis. Fatty acid methyl esters were separated on 70% (cyanopropyl-polysil phenylene siloxane) capillary column; injection temperature was set at 200 °C and detector temperature at 250 °C (MSD). The flow rate of the carrier, Helium, was 1.5 mL/min. The column temperature was 80 °C for 2 min then increased to 230 °C by the rate of 3 °C/min. For TLC, pre-coated silica gel 60 F-254 plates (Merck, Germany) were used, solvent systems were CHCl_3_ (100%), CHCl_3_/MeOH (95:5) and CHCl_3_/MeOH (85:15), spots were visualized by *p*-anisaldehyde/sulphuric acid reagent. Doxorubicin (Sigma-Aldrich, Darmstadt, Germany) was used as positive cytotoxic control.

### 3.2. Animal Material

The sponge was collected off Sharm El-Sheikh in the Red Sea at a depth of 10–14 m in July 2013. Identification of the sponge was kindly provided by Prof. Rob van Soest. The sponge consists of a group of thick-walled tubes with bluish green color in life, beige-yellow in preserved condition. The individual tubes measure up to 6 cm high and 2 cm in diameter, with a terminal oscule which measures up to 3 mm in preserved condition. The surface is optically smooth. The consistency is hard but toughly compressible. This species belongs to a group of “soft” lithistids. The skeleton is mainly a desma reticulation but in more than half the outer part of the tubes, the desmas are absent and the skeleton is there formed by bundles of strongyloxeas (strongyles with tapering endings) measuring 384–462 × 5–12 μm. The surface membrane lacks the usual phyllotriaenes but is crowded with acanthomicrorhabd microscleres measuring 9–13 × 2–3 μm, and these are densely distributed throughout the choanosome. In the inner parts of the tubes, single desmas gradually form a loose reticulation. The desmas are peculiar in being calthrops-like and having four bifurcate cladi with trilophose sharply pointed endings. The cladomes measure 240–270 μm. The primary cladi measure up to 100–120 × 20 μm, while the secondary cladi measure 30–35 × 15 μm. The specimen is assigned to the widespread Indo-Pacific species *Theonella aff. mirabilis*, because it conforms closely to its original description from Micronesia. In view of the great distance from the type locality, some doubt of conspecificity is here expressed. A voucher fragment was deposited in the Naturalis Biodiversity Center at Leiden under registration number ZMA Por. 17590. Another voucher was deposited at the Department of Natural Products at King Abdulaziz University under the code #DY-60.

### 3.3. Extraction and Isolation

The freeze-dried sponge (750 g) was extracted with a mixture of CH_2_Cl_2_/MeOH (1:1) (3 × 2000 mL) at room temperature. The combined extracts were concentrated under vacuum to afford 11.0 g of dried extract. The extract was partitioned between CHCl_3_ and H_2_O and the organic layer was evaporated under vacuum to afford 6.0 g of dried residue. The residue was then chromatographed on SiO_2_ VLC using *n*-hexane/EtOAc/MeOH gradient to give five main fractions (A–E). Fraction B (eluted by *n*-hexane/EtOAc mixture (70:30, 1.2 g) was purified on silica gel column using *n*-hexane/CHCl_3_ gradient to give several fractions. The active fraction was purified on C18-reversed phase semipreparative HPLC using 70% ACN to give compound **3** (4.9 mg). Fraction C which was eluted by *n*-hexane/EtOAc (60:40–20:80, 570 mg) was purified on silica gel column using *n*-hexane/CHCl_3_ gradient and final HPLC purification of the active fraction on C18-reversed phase semipreparative HPLC using 70% ACN to yield compound **4** (14 mg). Fraction D was eluted with EtOAc/MeOH (80:20–60:40, 930 mg) was subjected to SiO_2_ column using CHCl_3_/MeOH gradient to give four fractions. The cytotoxic fraction was subjected to final HPLC purification on C18 semipreparative column using 60% ACN to afford compound **1** (5.8 mg). Finally, fraction E was eluted with EtOAc/MeOH mixtures (40:60, 280 mg) was chromatographed over silica gel column using CHCl_3_/MeOH gradient to give several fractions. The cytotoxic fraction was purified on HPLC using 60% ACN to give compound **2** (3.3 mg).

### 3.4. Spectral Data of the Compounds

Mirabolide A (**1**). White precipitate; ${[\alpha ]}_{D}^{25}$ +0.36° (*c* 0.025, CH_3_OH); UV (MeOH) λ_max_ 292 nm; IR: ν_max_ 3350, 1735 cm^−1^; HRESIMS *m*/*z* 685.5625 (calcd. for C_40_H_77_O_8_, 685.5624 [M − H]^−^); NMR data: see Table 1.

Mirabolide B (**2**). White precipitate; ${[\alpha ]}_{D}^{25}$ −0.52° (*c* 0.025, CH_3_OH); UV (MeOH) λ_max_ 292 nm; IR: ν_max_ 3240, 1740 cm^−1^; HRESIMS *m*/*z* 713.5933 (calcd. for C_42_H_81_O_8_, 713.5931 [M + H]^+^); NMR data: see Table 2.

Conicasterol (**3**). White precipitate; ${[\alpha ]}_{D}^{25}$ +79° (*c* 0.1, CHCl_3_); UV (MeOH) λ_max_ 235 nm; IR: ν_max_ 3450, 1712 cm^−1^; ESIMS: *m*/*z* 413.3 [M + H]^+^, C_29_H_49_O.^1^H NMR (600 MHz, CDCl_3_): δ_H_ 5.07 (d, *J* = 1.2, H-29a), 4.63 (d, *J* = 0.6 Hz, H-29b), 4.02 (dd, *J* = 10.8, 4.8, H-3), 2.48 (ddd, H-15a), 2.25 (m, H_2_-7), 1.95 (dt, H-12b), 1.78 (brd, *J* = 14.4, H-5), 1.75 (m, H-1a), 1.75 (dd, *J* = 10.2, 2.4, H-9), 1.74 (m, H-15b), 1.64 (m, H-11a), 1.58 (m, H-16a), 1.49 (m, H-11b), 1.52 (m, H-25), 1.48 (m, H-6b), 1.45 (m, H-20), 1.39 (m, H-6a), 1.38 (m, H-16b), 1.35 (m, H-1b), 1.33 (m, H-22a), 1.23 (m, H_2_-2), 1.22 (m, H-23a), 1.22 (m, H-24), 1.14 (m, H-12a), 1.13 (m, H-17), 1.13 (m, H-22b), 1.10 (m, H-23b), 0.92 (d, *J* = 6.6, H_3_-21), 0.85 (d, *J* = 6.6, H_3_-27), 0.83 (s, H_3_-18), 0.80 (d, *J* = 7.2, H_3_-26), 0.78 (d, *J* = 6.6, H_3_-28), 0.58 (s, H_3_-19); ^13^C NMR (150 MHz, CDCl_3_): δ_C_ 153.1 (C-4), 142.9 (C-14), 125.6 (C-8), 102.7 (C-29), 73.3 (C-3), 56.8 (C-17), 49.4 (C-5), 49.2 (C-9), 42.7 (C-13), 39.9 (C-10), 37.3 (C-24), 36.7 (C-12), 34.5 (C-1), 32.3 (C-20), 31.9 (C-22), 30.1 (C-25), 29.3 (C-23), 27.1 (C-2), 27.0 (C-15), 25.7 (C-6), 24.6 (C-7), 22.7 (C-16), 20.2 (C-11), 19.0 (C-27), 18.2 (C-21), 18.1 (C-26), 15.3 (C-18), 14.1 (C-28), 13.1 (C-19).

Swinhosterol B (**4**). White precipitate; ${[\alpha ]}_{D}^{25}$ −50° (*c* 0.1, CHCl_3_); UV (MeOH) λ_max_ 280 nm; IR: ν_max_ 3460, 1735, 1710 cm^−1^; ESIMS: *m*/*z* 445.4 [M + H]^+^, C_29_H_49_O_3_.^1^H NMR (600 MHz, CDCl_3_): δ_H_ 5.16 (brs, H-29b), 4.67 (brs, H-29a), 4.0 (dd, *J* = 11.7, 5.9, H-3), 2.41 (m, H_2_-7), 2.35 (m, H-15b), 2.29 (m, H-5), 2.20 (m, H-16b), 2.12 (m, H-9), 2.10 (m, H-2b), 2.04 (m, H-15a), 1.97 (m, H-17), 1.97 (m, H-6b), 1.82 (m, H-6a), 1.77 (m, H-1b), 1.71 (m, H-11b), 1.63 (m, H-12b), 1.53 (m, H-25), 1.51 (m, H-20), 1.48 (m, H-1a), 1.47 (m, H-16a), 1.43 (m, H-22a), 1.41 (m, H-12a), 1.37 (m, H-22b), 1.29 (m, H-2a), 1.25 (m, H-23b), 1.23 (m, H-24), 1.19 (m, H-23a), 1.08 (d, *J* = 6.6, H_3_-21), 0.87 (d, *J* = 6.6, H_3_-27), 0.82 (d, *J* = 6.6, H_3_-26), 0.81 (d, *J* = 5.1, H_3_-28), 0.81 (m, H-11a); ^13^C NMR (150 MHz, CDCl_3_): δ_C_ 224.9 (C-14), 211.2 (C-8), 150.9 (C-4), 104.2 (C-29), 72.9 (C-3), 62.5 (C-9), 52.5 (C-13), 48.4 (C-5), 46.6 (C-17), 44.3 (C-10), 41.6 (C-7), 38.9 (C-24), 37.9 (C-15), 37.3 (C-12), 36.5 (C-1), 34.4 (C-20), 32.4 (C-25), 32.3 (C-22), 32.2 (C-2), 30.6 (C-23), 26.0 (C-6), 23.7 (C-16), 20.2 (C-27), 18.5 (C-18), 18.2 (C-26), 18.1 (C-21), 18.0 (C-11), 15.4 (C-28), 13.0 (C-19).

### 3.5. Methanolysis of Compounds **1** and **2** and Methylation of Fatty Acids

Compounds **1** and **2** (3 mg, each) were separately refluxed with 5% methanolic KOH (5 mL) for 2 h [29]. The reaction product was diluted with water and extracted with CHCl_3_. The fatty acids were liberated from the aqueous layer after acidification with 1 N HCl followed by extraction with CHCl_3_, then were methylated using CH_2_N_2_ and subjected to GC-MS analysis.

### 3.6. Evaluation of the Cytotoxic Activities of Compounds **1**–**4**

The cytotoxic activity of compounds **1**–**4** against breast adenocarcinoma cell line (MCF-7, ATCC^®^ HTB-22™) were evaluated using sulforhodamine assay [30]. Cells were grown 24 h in 96-well plates before addition of samples, then incubated for 48 h after treatment with tested compounds. The dose of the compound that reduced survival to 50% (IC_50_) was calculated from the log dose response curve. The values were the results of three determinations. Doxorubicin was used as reference drug and showed an IC_50_ value of 5.4 μg/mL.

## 4. Conclusions

Column chromatographic fractionation of the organic extract of the Red Sea sponge *Theonella mirabilis* afforded four compounds (**1**–**4**) including two sterols and two glycerides. Compounds **1** and **2** are new glycerides and are reported here for the first time. The structures of the isolated compounds were determined by careful examination of their 1D and 2D NMR, and HRESIMS spectral data. All isolated compounds demonstrated significant cytotoxic activity against human breast adenocarcinoma cell line (MCF-7) with IC_50_ values ranging from 3.0 to 16.4 μg/mL.

## Figures and Tables

**Table 1:** NMR data of compound **1** (CDCl_3_, 600 & 150 MHz).

Position	δ_C_ (mult.) ^a^	δ_H_ (mult., *J* in Hz)	HMBC (H→C) ^b^
**1**	67.7 CH_2_	3.78, dd (10.8, 4.2) 3.73, dd (10.8, 6.6)	H-2, H_2_-3, H-1′
**2**	69.3 CH	5.17, quin (5.4)	H_2_-1, H_2_-3
**3**	67.1 CH_2_	3.57, dd (10.8, 5.4) 3.54, dd (10.8, 4.8)	H_2_-1, H-2, H_2_-1′′′, H_2_-2′′′
**1′**	80.3 CH	3.66, t (6.0)	H_2_-1, H-2′, H-3′, H-4′, H-5′
**2′**	71.4 CH	3.93, t (6.0)	H-1′, H-3′, H-4′, H-5′
**3′**	78.5 CH	3.84, t (6.0)	H-1′, H-2′, H-4′, H-5′
**4′**	77.0 CH	3.69, t (7.2)	H-1′, H-2′, H-3′, H-5′
**5′**	77.2 CH	3.90, t (6.6)	H-1′, H-2′, H-3′, H-4′
**1″**	172.1 qC	-	H-2, H-1′, H_2_-2″, H_2_-3″
**2″**	32.5 CH_2_	2.33, t (7.8)	H_2_-3″
**3″**	23.0 CH_2_	1.60, quin (7.2)	H_2_-2″
**4″–11″**	20.8–35.1 CH_2_	1.25, m	
**12″**	35.2 CH_2_	1.06, m	H_2_-11″, H-13″, H_3_-16″
**13″**	30.8 CH	1.35, m	H_2_-12″, H_3_-15″, H_3_-16″
**14″**	30.0 CH_2_	1.25, m	H-13″, H_3_-15″, H_3_-16″
**15″**	12.2 CH_3_	0.88, t (6.6)	H-13″, H_2_-14″
**16″**	17.7 CH_3_	0.83, d (6.6)	H_2_-12″, H-13″, H_2_-14″
**1′′′**	70.0 CH_2_	3.45, dd (6.6, 2.4) 3.41, dd (6.6, 2.4)	H_2_-3′′′, H_2_-2′′′
**2′′′**	27.8 CH_2_	1.53, quin (7.2)	H_2_-1′′′, H_2_-3′′′
**3′′′–12′′′**	20.8–35.1 CH_2_	1.25, m	
**13′′′**	37.1 CH_2_	1.15, m	H_2_-12′′′, H_3_-15′′′, H_3_-16′′′
**14′′′**	26.0 CH	1.50, m	H_2_-13′′′, H_3_-15′′′, H_3_-16′′′
**15′′′**	20.7 CH_3_	0.85, d (6.6)	H_2_-12′′′, H_2_-13′′′, H-14′′′, H_3_-16′′′
**16′′′**	20.7 CH_3_	0.85, d (6.6)	H_2_-12′′′, H_2_-13′′′, H-14′′′, H_3_-15′′′

^a^ multiplicities were deduced from DEPT and multiplicity-edited HSQC; ^b^ HMBC correlations are from proton(s) stated to the indicated carbons.

**Table 2:** NMR data of compound **2** (CD_3_OD, 600 & 150 MHz).

Position	δ_C_ (mult.) ^a^	δ_H_ (mult., *J* in Hz)	HMBC (H→C) ^b^
**1**	70.5 CH_2_	3.60, m	H-2, H_2_-3
**2**	71.5 CH	5.22, m	H_2_-1, H_2_-3
**3**	63.9 CH_2_	4.40, dd (12.0, 3.0) 4.16, dd (12.0, 7.2)	H_2_-1, H-2
**1′**	175.1 qC	-	H_2_-1, H_2_-2′, H_2_-3′
**2′**	35.0 CH_2_	2.30, m	H_2_-3′, H_2_-4′
**3′**	26.1 CH_2_	1.59, m	H_2_-2′, H_2_-4′
**4′–11′**	28.2–31.1 CH_2_	1.28, m	
**12′**	38.2 CH_2_	1.10, m	H_2_-11′, H-13′, H_2_-14′, H_3_-16′
**13′**	35.7 CH	1.31, m	H_2_-12′, H_3_-15′, H_3_-16′
**14′**	30.8 CH_2_	1.28, m	H_2_-12′, H-13′, H_3_-15′, H_3_-16′
**15′**	11.8 CH_3_	0.88, t (6.6	H-13′, H_2_-14′
**16′**	19.6 CH_3_	0.85, d (6.6)	H_2_-12′, H-13′, H_2_-14′
**1″**	171.6 qC	-	H-2″, H_2_-3″, H_2_-4″
**2″**	77.5 CH	3.70, brd (3.0)	MeO-2″, H_2_-3″, H_2_-4″
**3″**	29.1 CH_2_	2.08, m; 2.22, m	H-2″, H_2_-4″
**4′′′**	68.6 CH_2_	3.53, m; 3.67, m	H- 2″, H_2_-3″, OCH_3_-2″
***Me*O-2″**	52.4 CH_3_	3.20, s	H-2″
**1′′′**	174.7 qC	-	H_2_-3, H_2_-2′′′, H_2_-3′′′
**2′′′**	35.1 CH_2_	2.30, m	H_2_-3′′′, H_2_-4′′′
**3′′′**	26.1 CH_2_	1.59, m	H_2_-2′′′, H_2_-4′′′
**4′′′–14′′′**	28.2–31.1 CH_2_	1.28, m	
**15′′′**	40.2 CH_2_	1.19, m	H_2_-14′′′, H-16′′′, H_3_-17′′′, H_3_-18′′′
**16′′′**	29.1 CH	1.50, m	H_3_-17′′′, H_3_-18′′′
**17′′′**	23.1 CH_3_	0.87, d (6.6)	H_2_-14′′′, H_2_-15′′′, H-16′′′
**18′′′**	23.1 CH_3_	0.87, d (6.6)	H_2_-14′′′, H_2_-15′′′, H-16′′′

^a^ multiplicities were deduced from DEPT and multiplicity-edited HSQC; ^b^ HMBC correlations are from proton(s) stated to the indicated carbons.

## Supplementary Materials

The followings are available online at www.mdpi.com/1660-3397/14/8/155/s1, Figure S1: ^1^H NMR Spectrum of **1** (CDCl_3_), Figure S2: ^13^C NMR Spectrum of **1** (CDCl_3_), Figure S3: COSY Spectrum of **1** (CDCl_3_), Figure S4: HSQC Spectrum of **1** (CDCl_3_), Figure S5: HMBC Spectrum of **1** (CDCl_3_), Figure S6: Partial HMBC Spectrum of **1** “Expansion A” (CDCl_3_), Figure S7: Partial HMBC Spectrum of **1** “Expansion B” (CDCl_3_), Figure S8: Partial HMBC Spectrum of **1** “Expansion C” (CDCl_3_), Figure S9: ROESY Spectrum of **1** (CDCl_3_), Figure S10: ^1^H NMR Spectrum of **2** (CD_3_OD), Figure S11: ^13^C NMR Spectrum of **2** (CD_3_OD), Figure S12: COSY Spectrum of **2** (CD_3_OD), Figure S13: HSQC Spectrum of **2** (CD_3_OD), Figure S14: HMBC Spectrum of **2** (CD_3_OD), Figure S15: Partial HMBC Spectrum of **2** “Expansion A” (CD_3_OD), Figure S16: Partial HMBC Spectrum of **2** “Expansion B” (CD_3_OD, Figure S17: Partial HMBC Spectrum of **2** “Expansion C” (CD_3_OD).
