# Supplementary material for: Mirabolides A and B; New Cytotoxic Glycerides from the Red Sea Sponge Theonella mirabilis
Source: Mar Drugs. 2016 Aug 18;14(8):155. doi: 10.3390/md14080155 (PMC4999916; doi:10.3390/md14080155)
Supplement: Supplementary file 1 [file marinedrugs-14-00155-s001.pdf]

Supplementary Materials: Mirabolides A and B; New Cytotoxic Glycerides from the Red Sea Sponge *Theonella mirabilis*

Dina R. Abou-Hussein and Diaa T. A. Youssef

Table Contents

**Figure S1**. ^{1}H NMR Spectrum of **1** ($CDCl_3$)	S2
**Figure S2.** ^{13}C NMR Spectrum of **1** ($CDCl_3$)	S3
**Figure S3.** COSY Spectrum of **1** ($CDCl_3$)	S4
**Figure S4.** HSQC Spectrum of **1** ($CDCl_3$)	S5
**Figure S5.** HMBC Spectrum of **1** ($CDCl_3$)	S6
**Figure S6.** Partial HMBC Spectrum of **1** "Expansion A" ($CDCl_3$)	S7
**Figure S7.** Partial HMBC Spectrum of **1** "Expansion B" ($CDCl_3$)	S8
**Figure S8.** Partial HMBC Spectrum of **1** "Expansion C" ($CDCl_3$)	S9
**Figure S9.** ROESY Spectrum of **1** ($CDCl_3$)	S10
**Figure S10.** ^{1}H NMR Spectrum of **2** (CD_3OD)	S11
**Figure S11.** ^{13}C NMR Spectrum of **2** (CD_3OD)	S12
**Figure S12.** COSY Spectrum of **2** (CD_3OD)	S13
**Figure S13.** HSQC Spectrum of **2** (CD_3OD)	S14
**Figure S14.** HMBC Spectrum of **2** (CD_3OD)	S15
**Figure S15.** Partial HMBC Spectrum of **2** "Expansion A" (CD_3OD)	S16
**Figure S16.** Partial HMBC Spectrum of **2** "Expansion B" (CD_3OD)	S17
**Figure S17.** Partial HMBC Spectrum of **2** "Expansion C" (CD_3OD)	S18

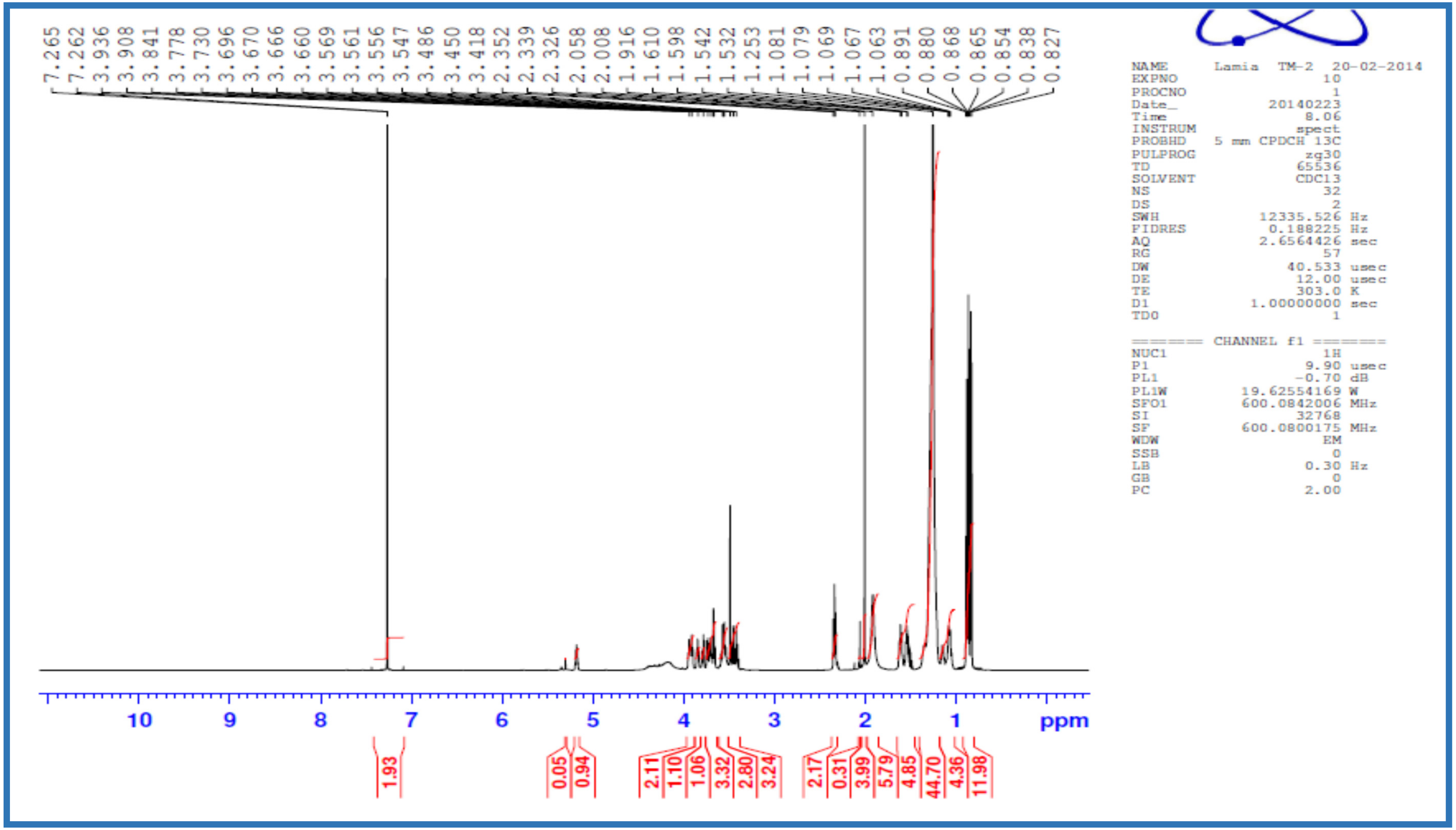

Figure S1. ^{1}H NMR Spectrum of **1** ($CDCl_3$).

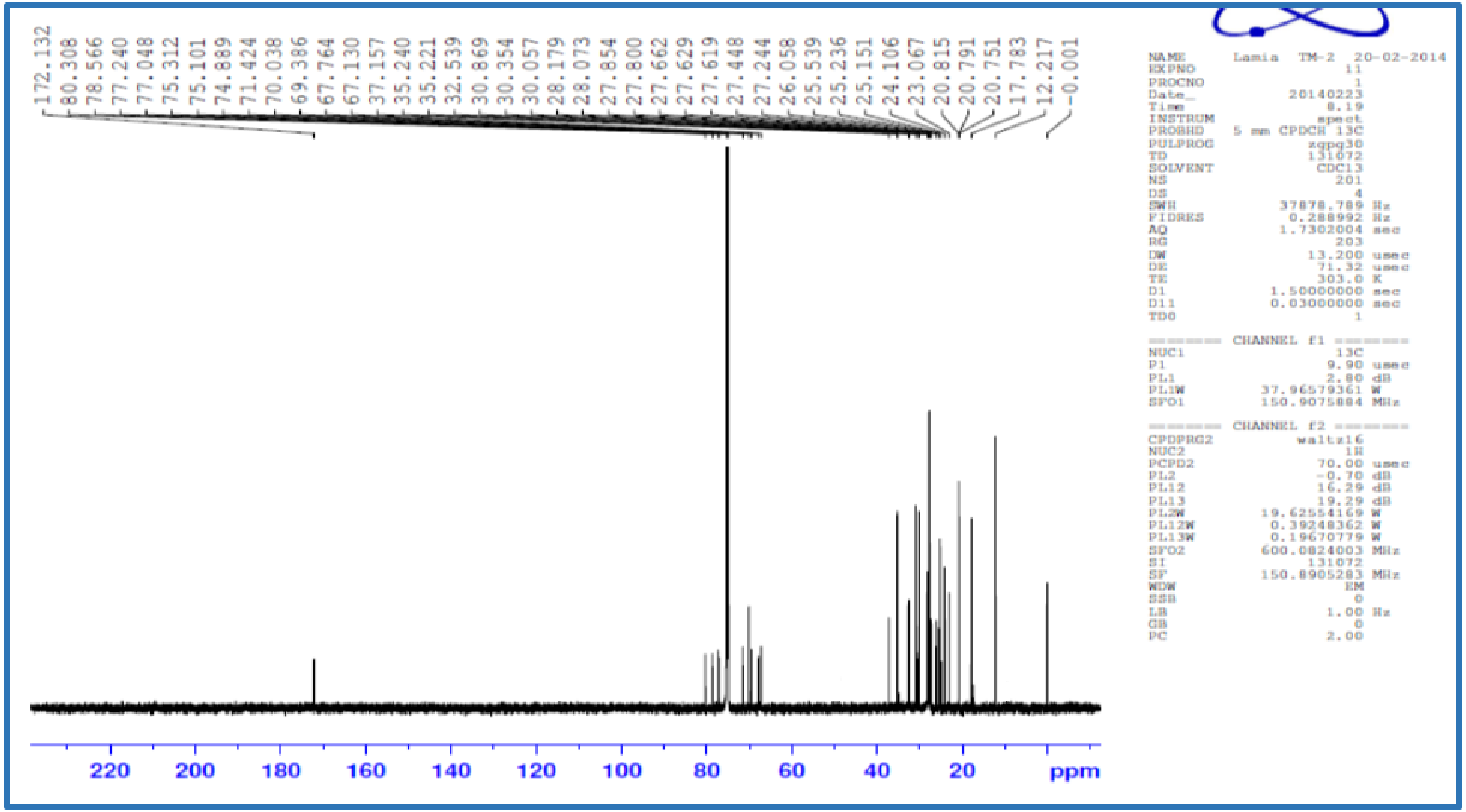

Figure S2. ^{13}C NMR Spectrum of **1** ($CDCl_3$).

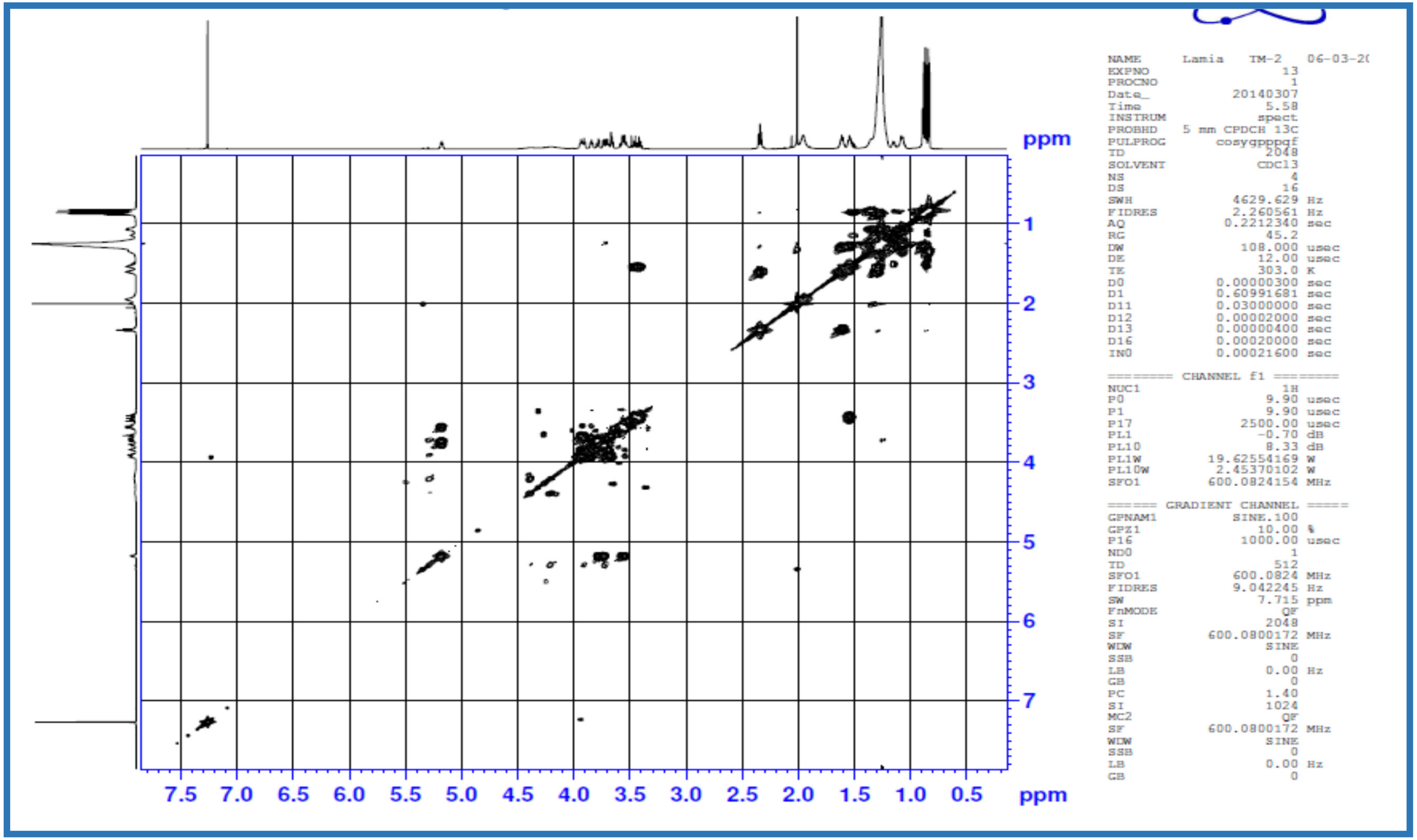

Figure S3. COSY Spectrum of **1** ($CDCl_3$).

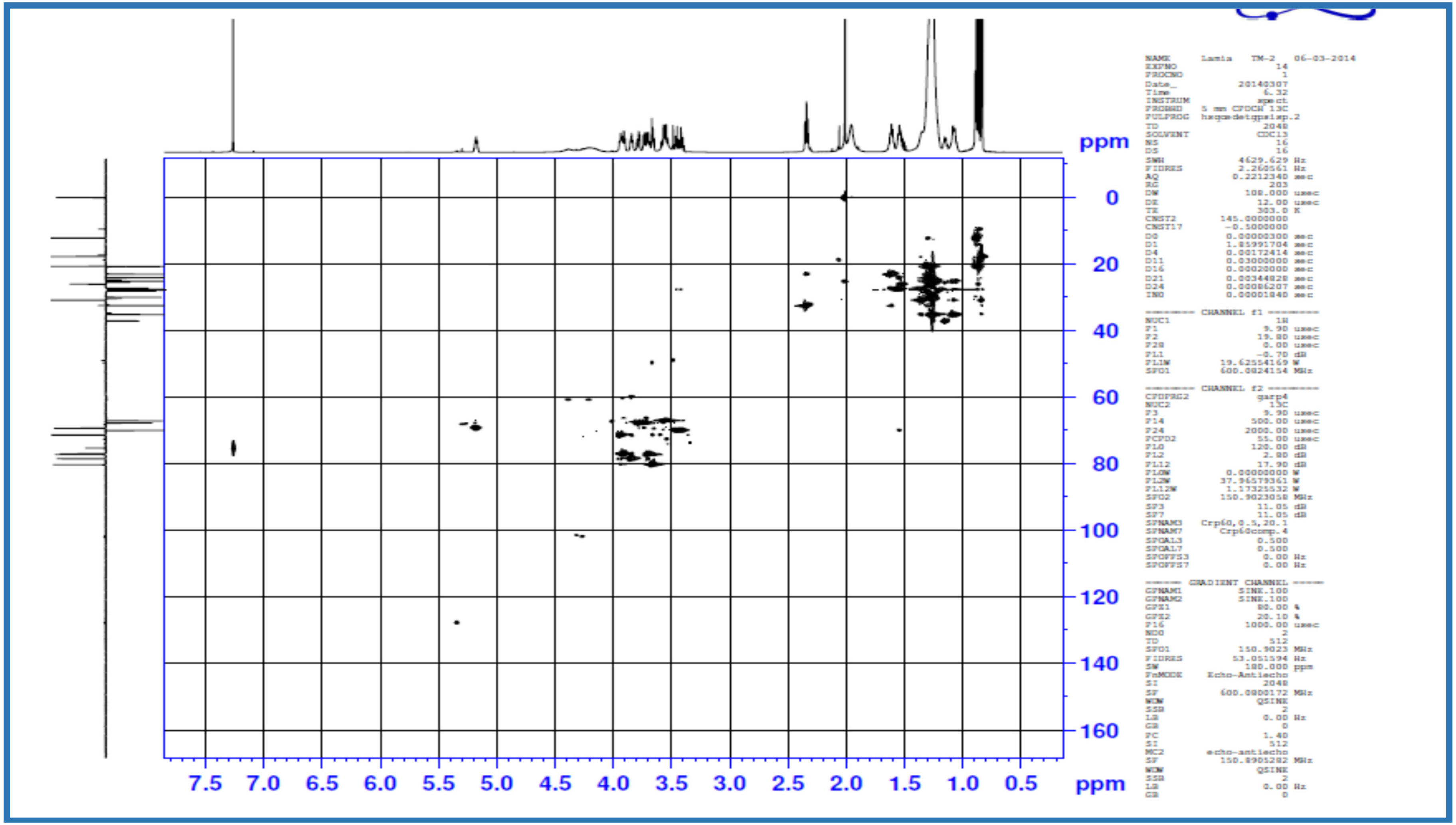

Figure S4. HSQC Spectrum of **1** ($CDCl_3$).

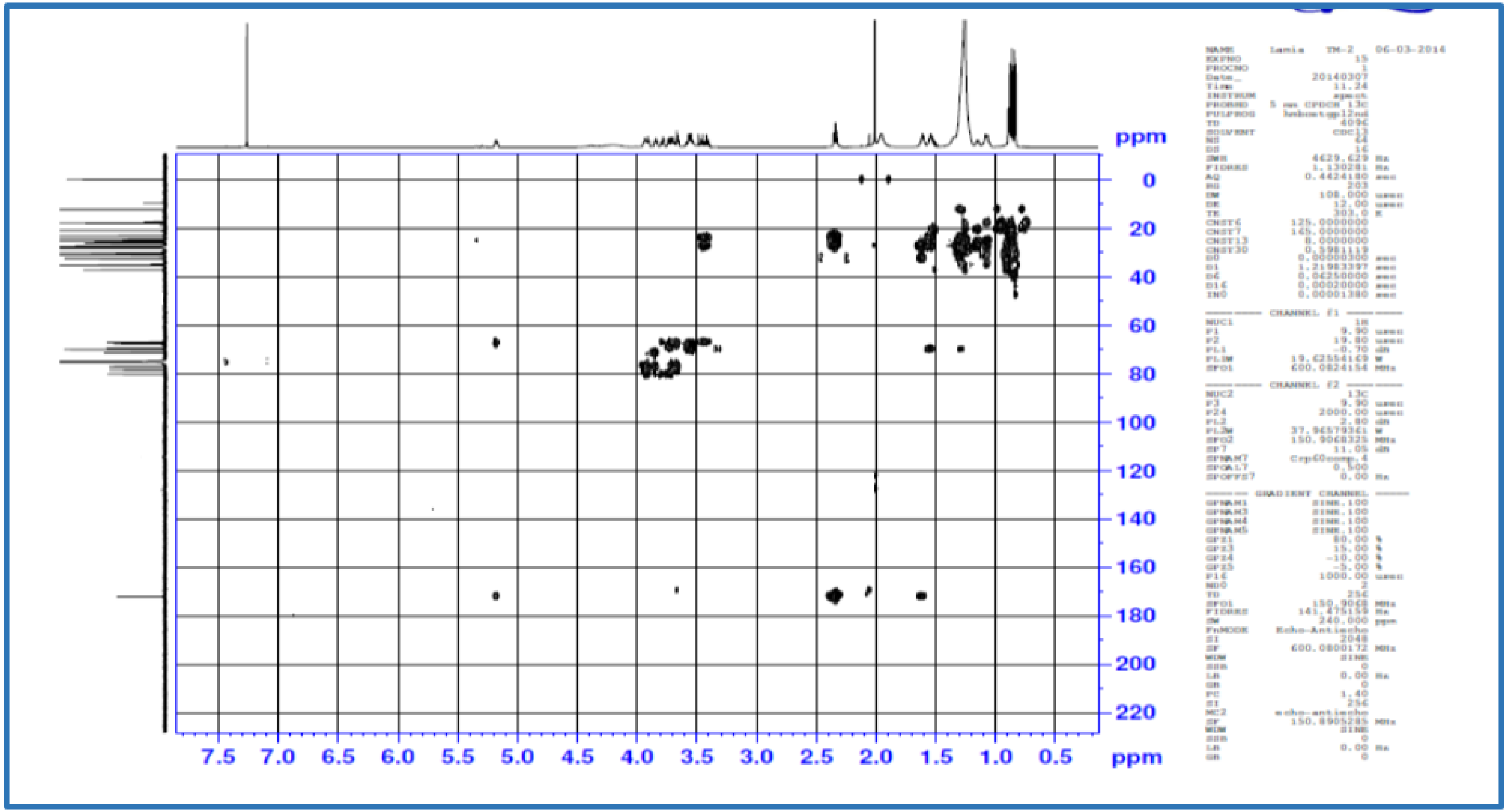

Figure S5. HMBC Spectrum of **1** ($CDCl_3$).

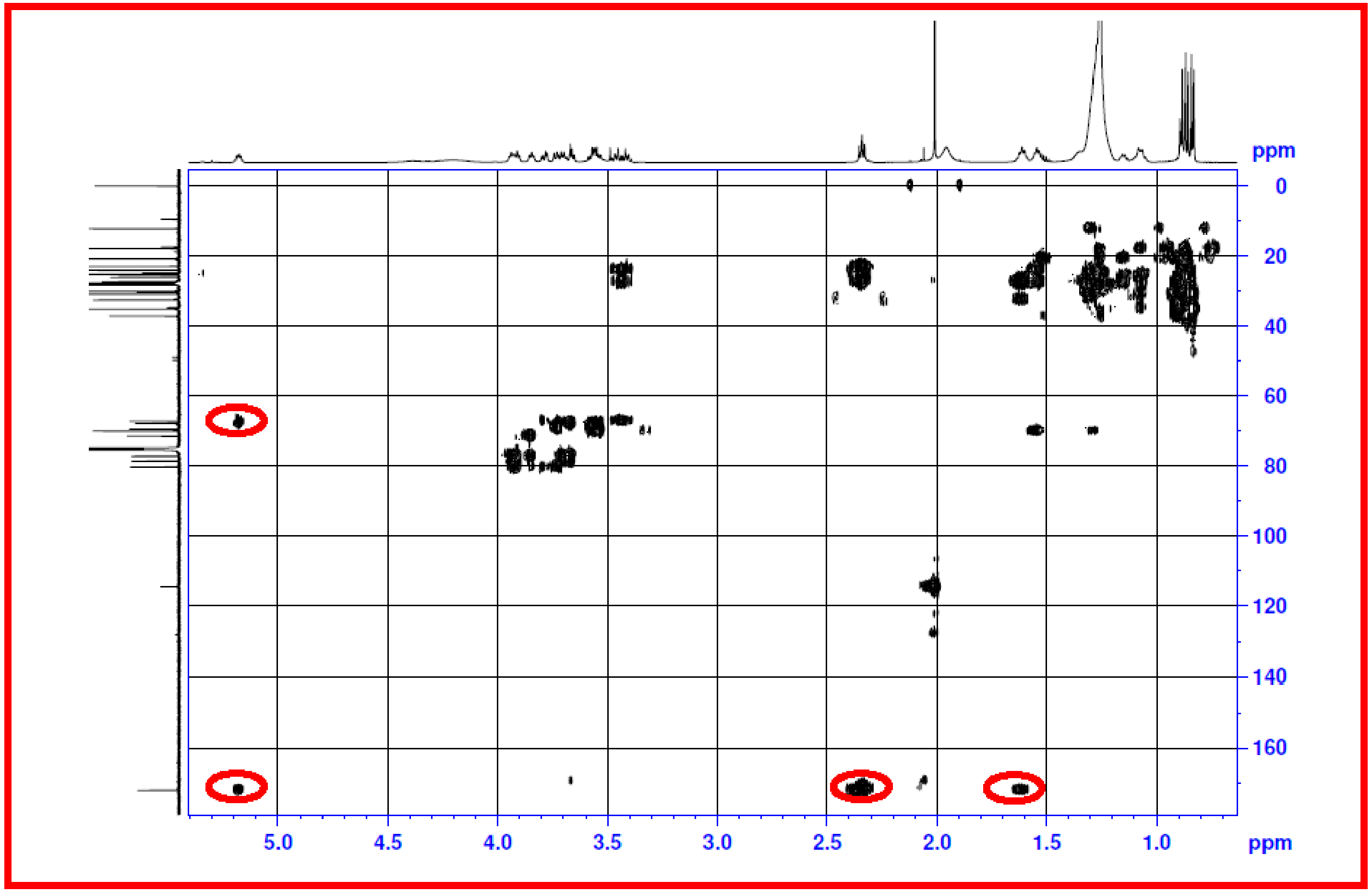

Figure S6. Partial HMBC Spectrum of **1** "Expansion A" ($CDCl_3$).

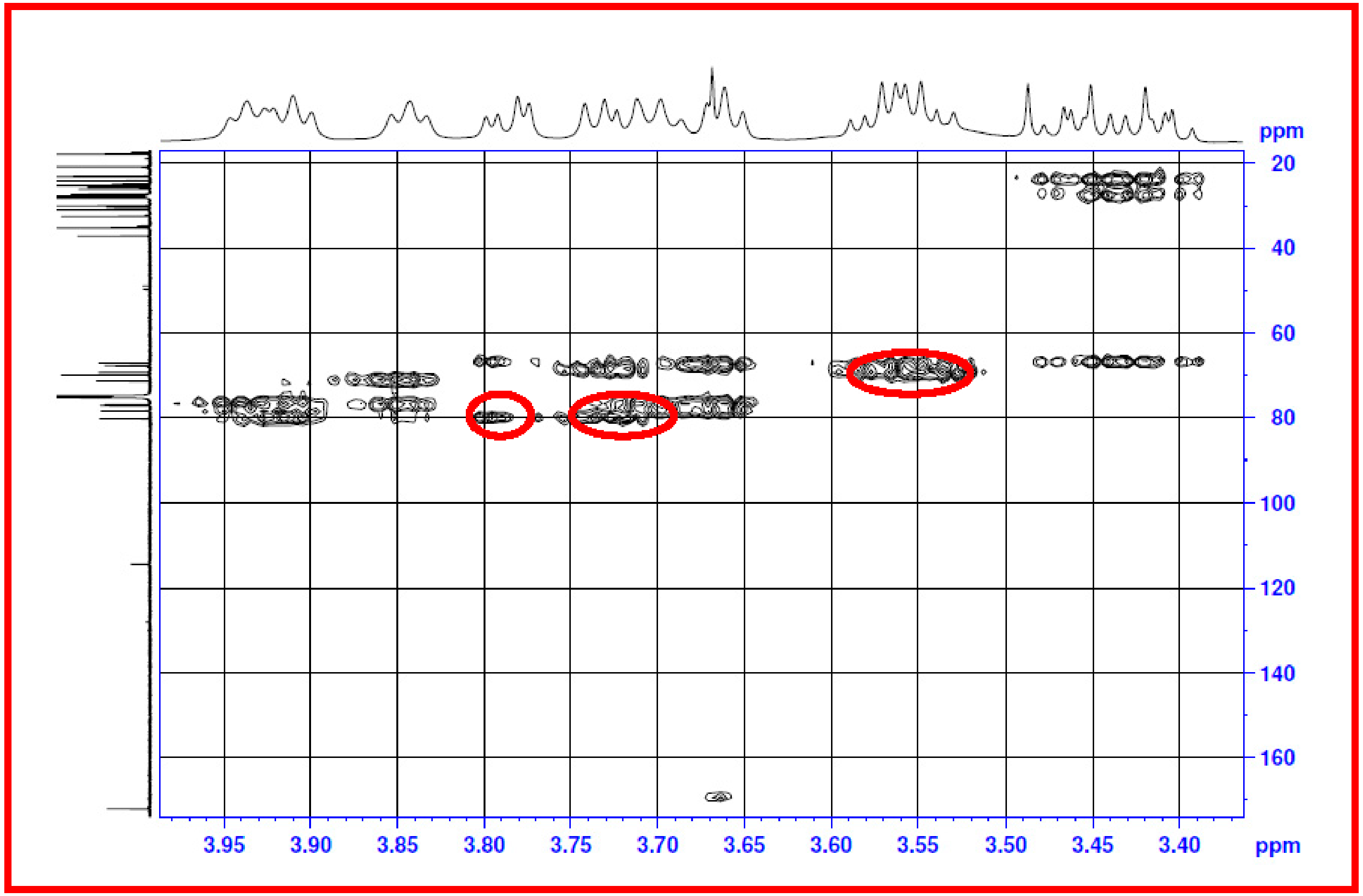

Figure S7. Partial HMBC Spectrum of **1** "Expansion B" ($CDCl_3$).

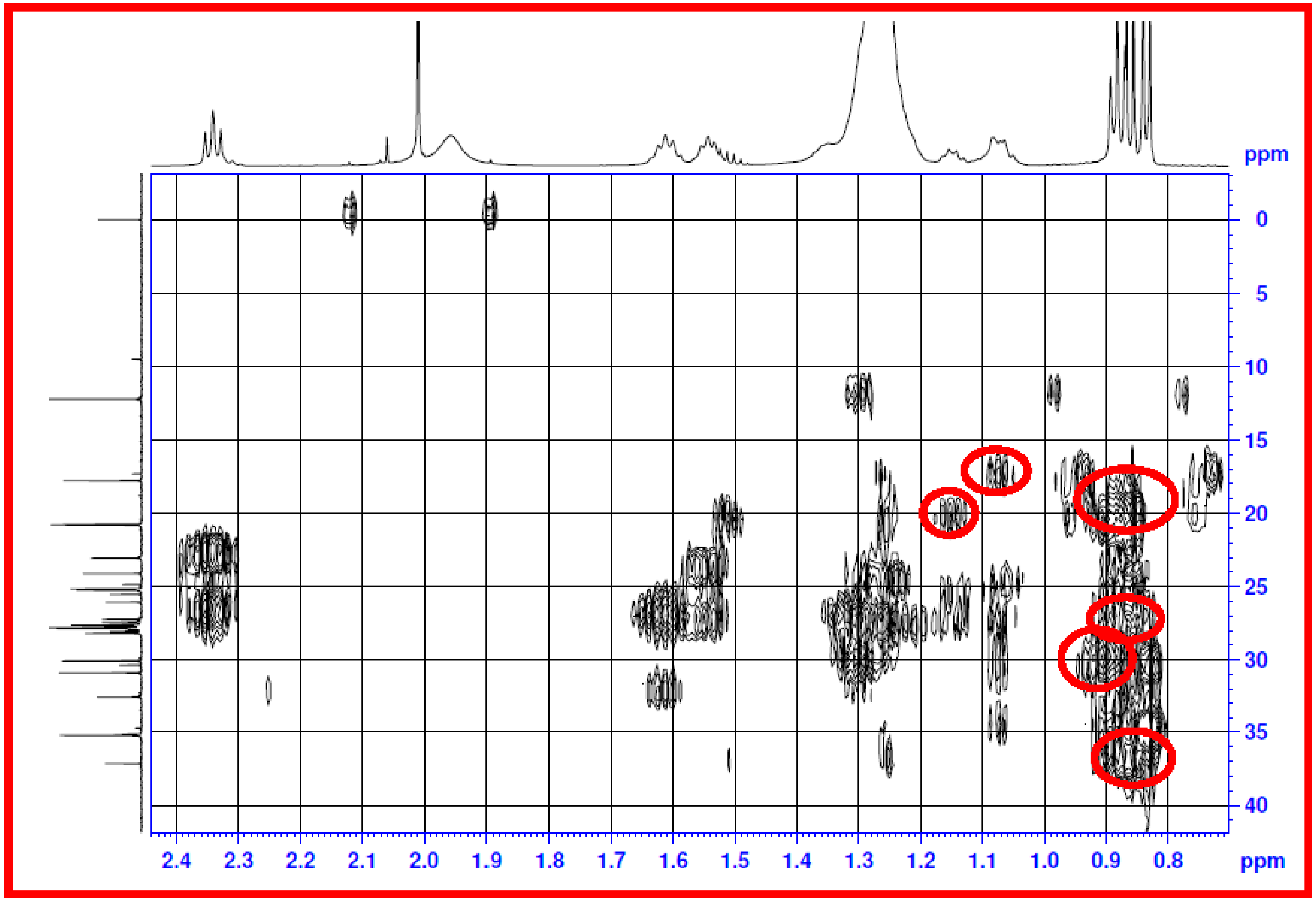

Figure S8. Partial HMBC Spectrum of **1** “Expansion C” ($CDCl_3$).

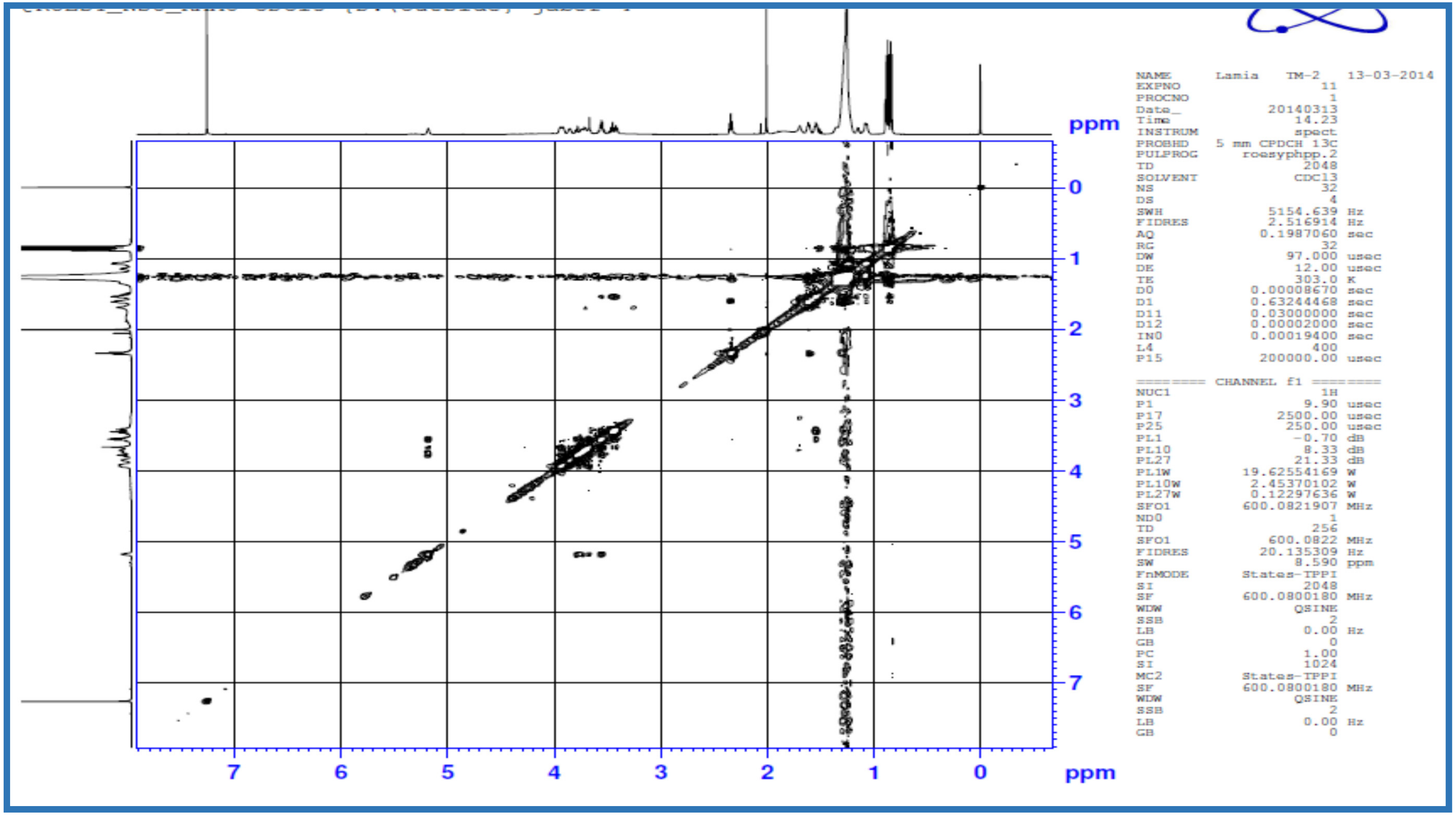

Figure S9. ROESY Spectrum of **1** ($CDCl_3$).

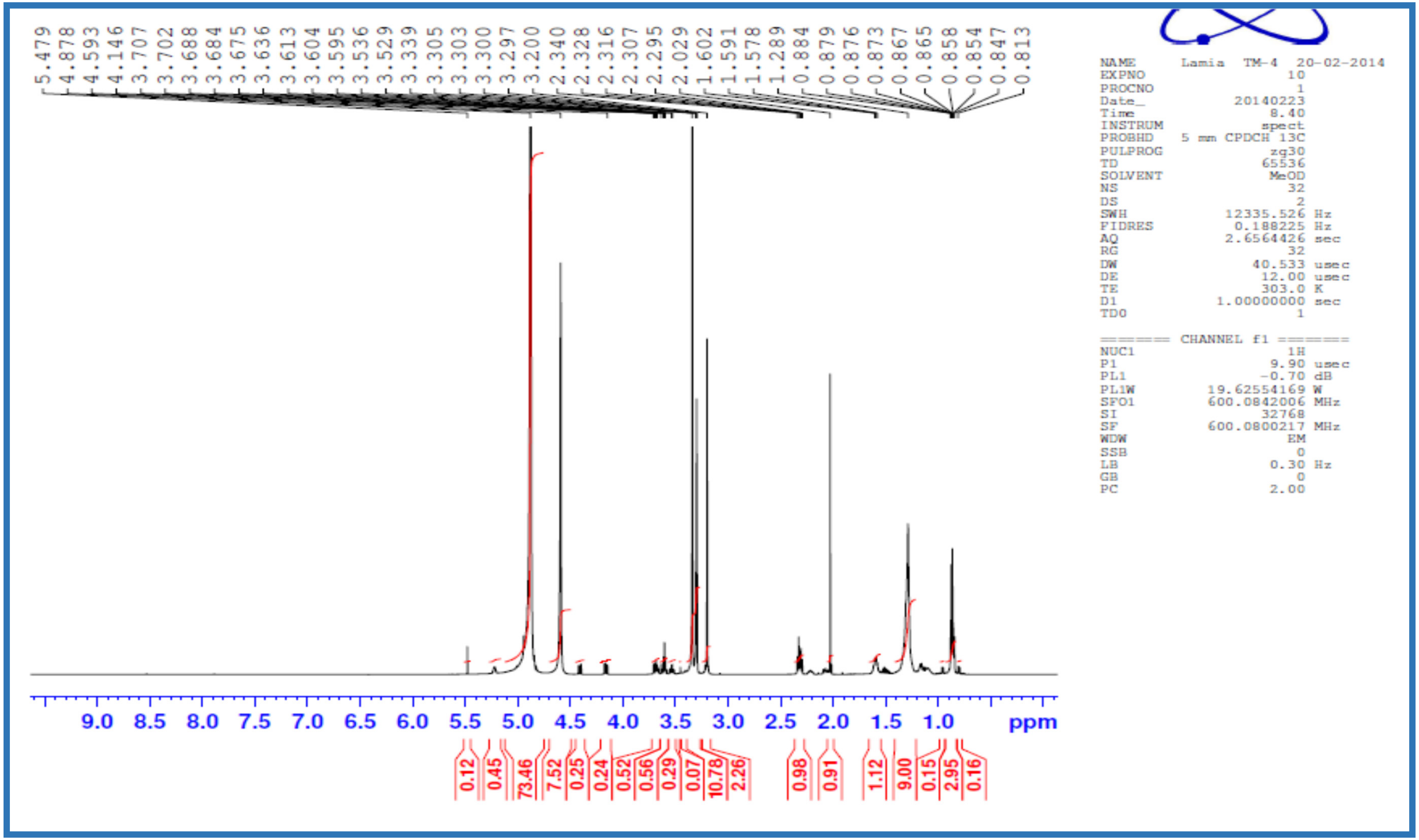

Figure S10. ^{1}H NMR Spectrum of **2** (CD_3OD).

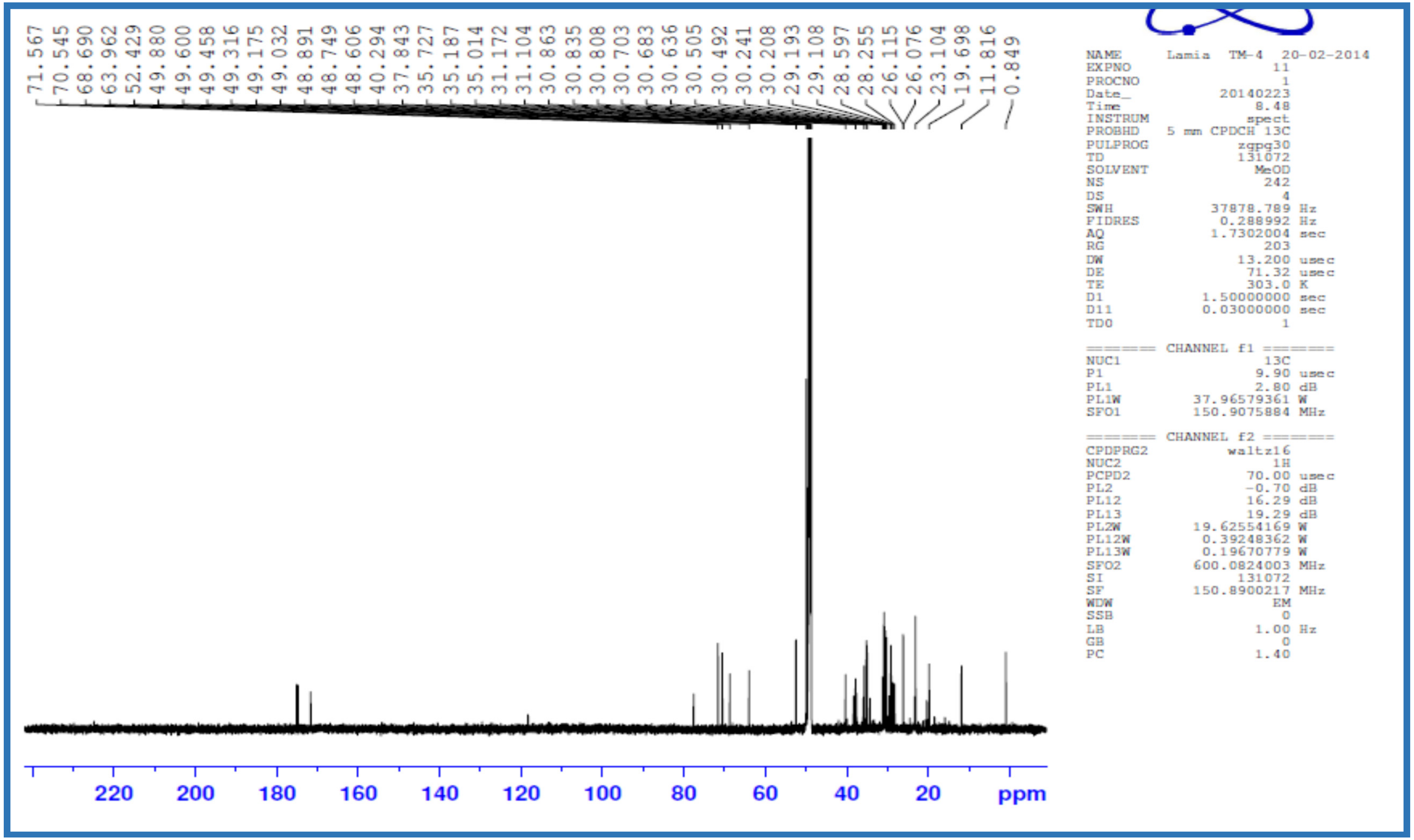

Figure S11. ^{13}C NMR Spectrum of **2** (CD_3OD).

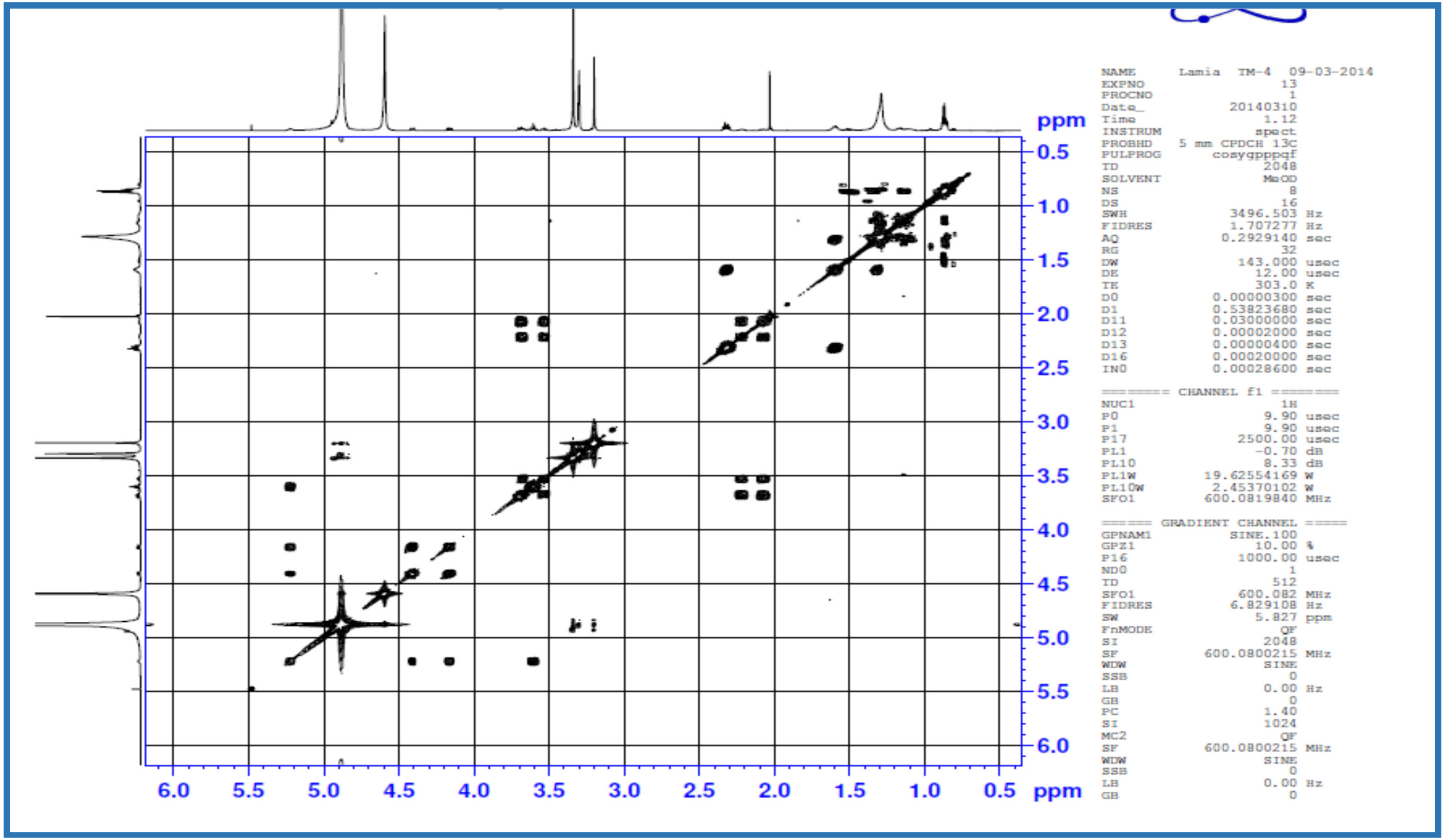

Figure S12. COSY Spectrum of **2** (CD_3OD).

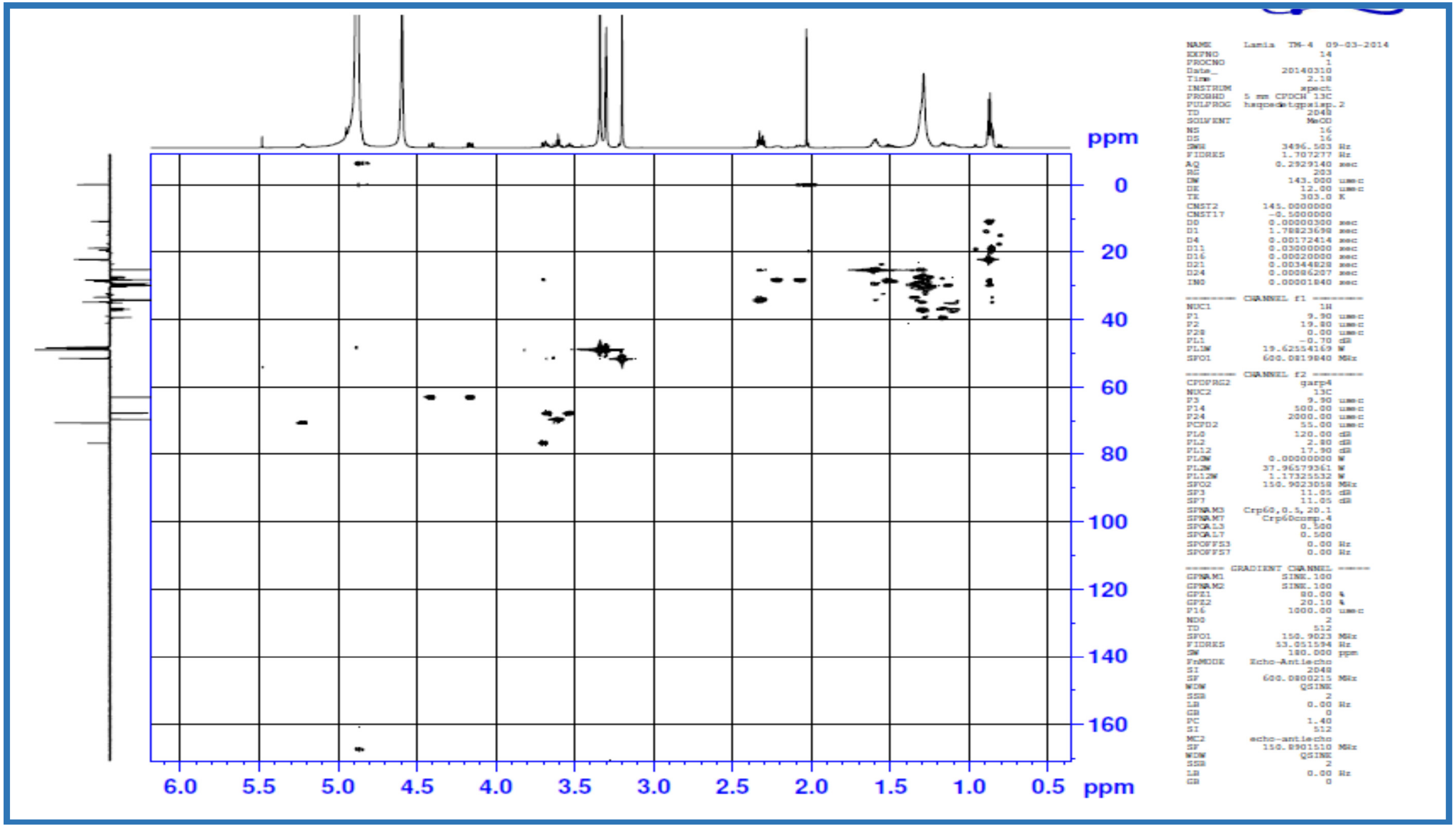

Figure S13. HSQC Spectrum of **2** (CD_3OD).

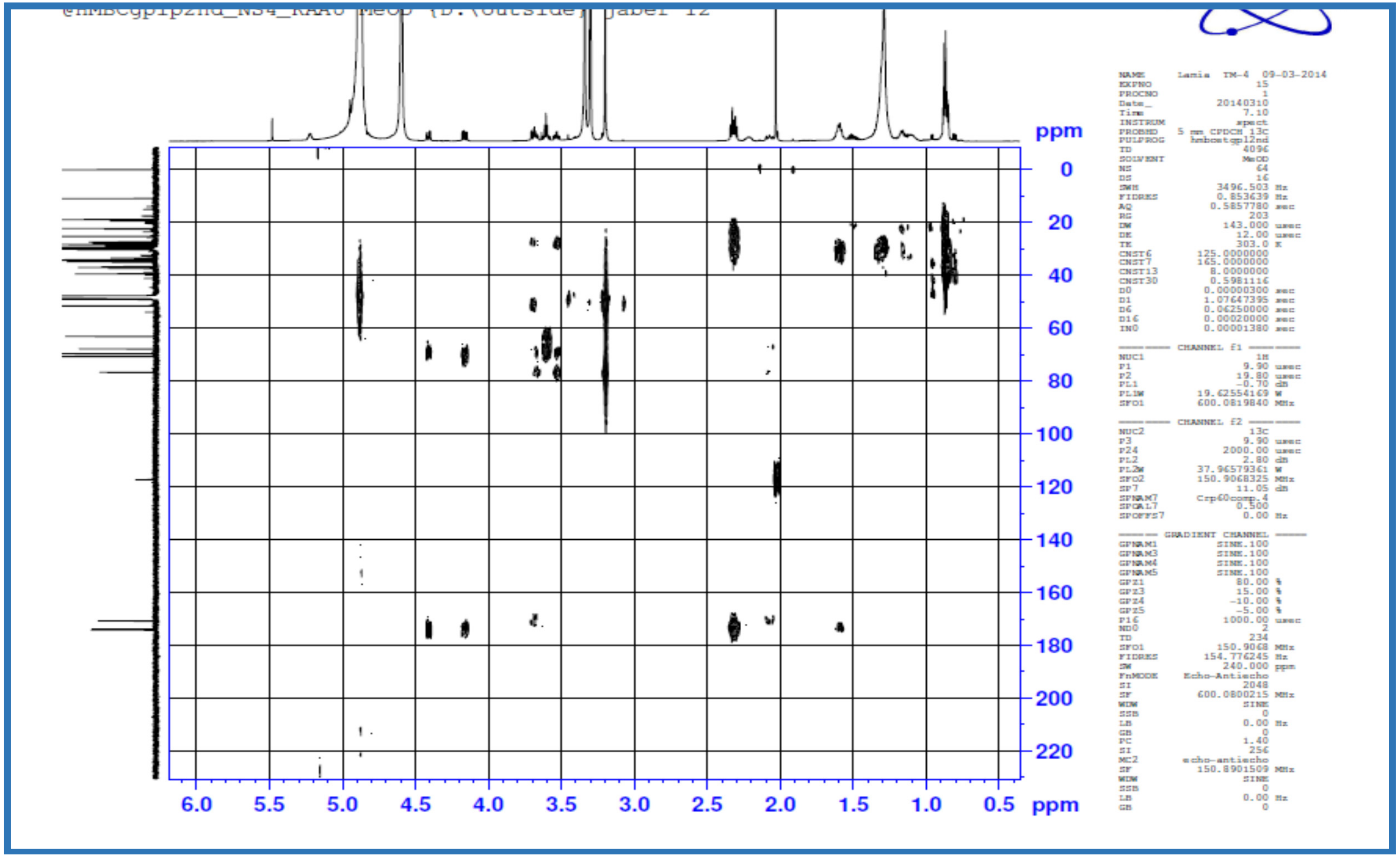

Figure S14. HMBC Spectrum of **2** (CD_3OD).

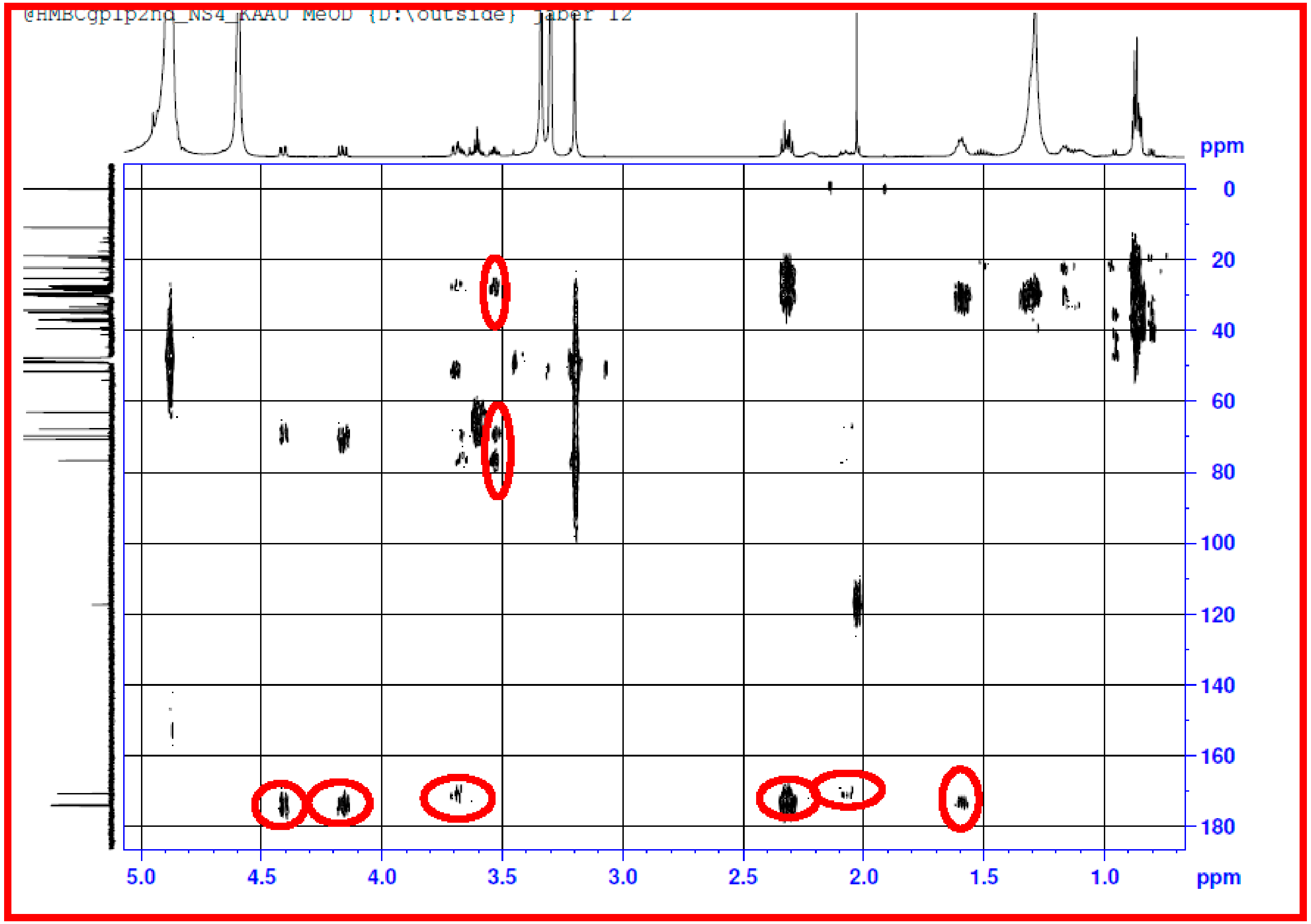

Figure S15. Partial HMBC Spectrum of **2** "Expansion A" (CD_3OD).

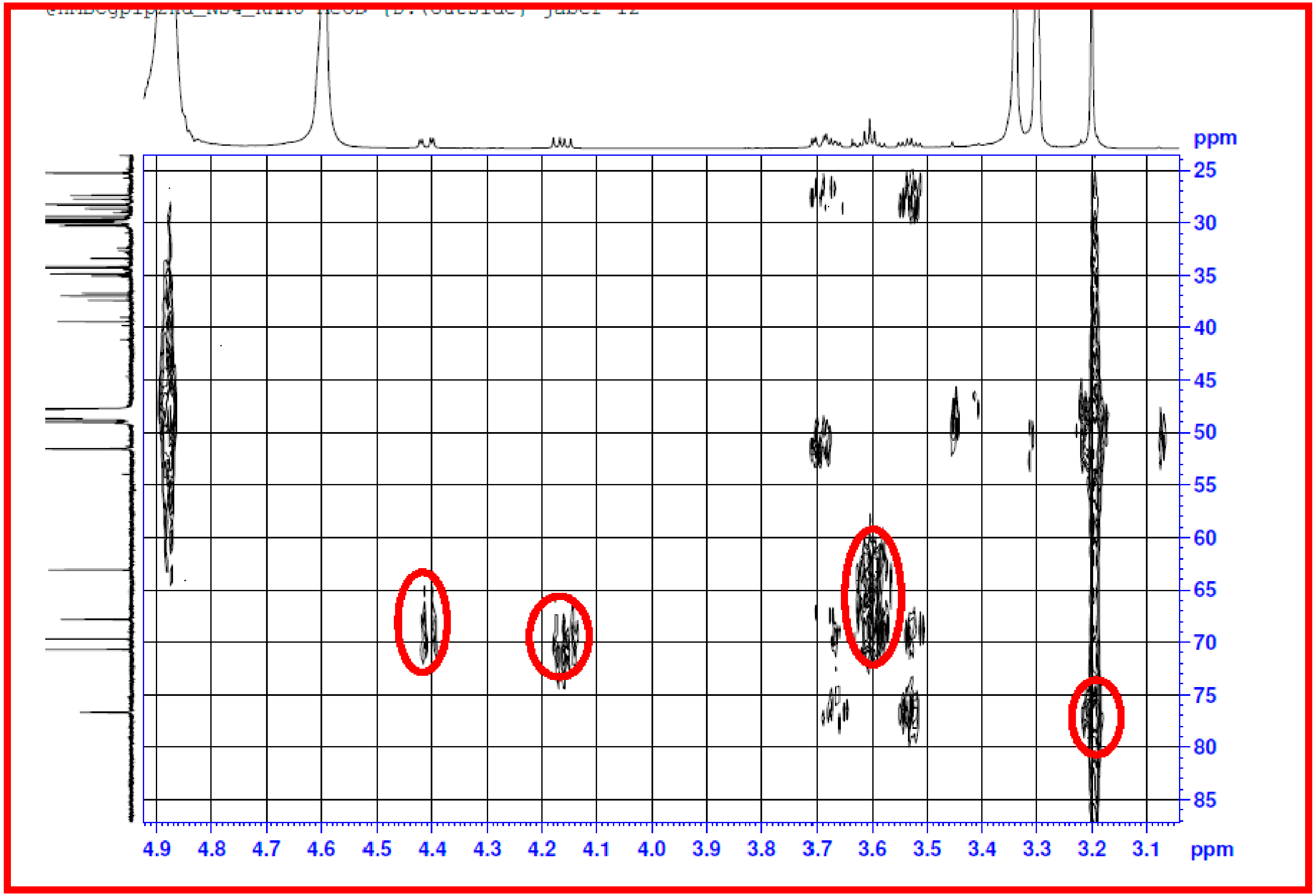

Figure S16. Partial HMBC Spectrum of **2** "Expansion B" (CD_3OD).

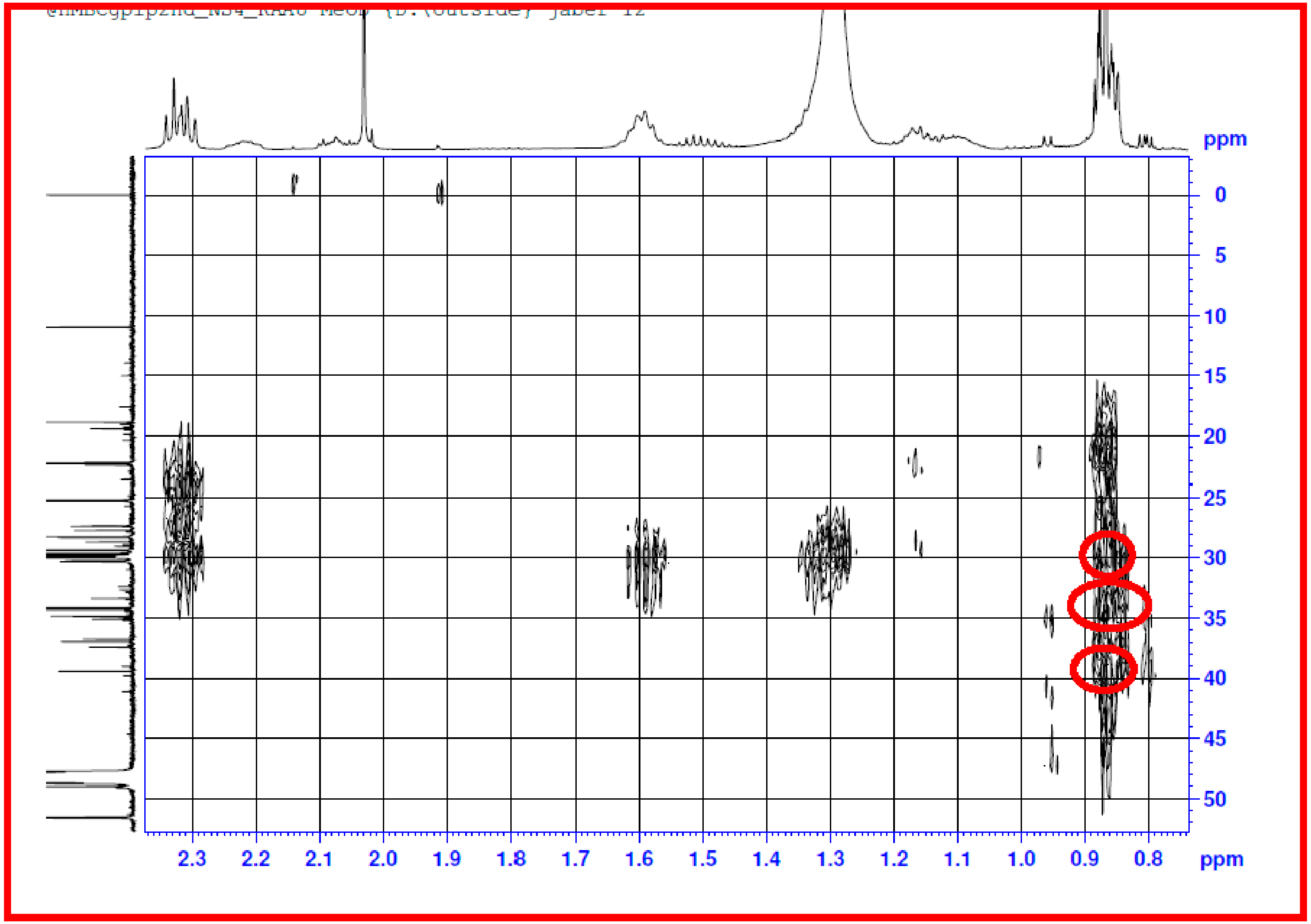

Figure S17. Partial HMBC Spectrum of **2** "Expansion C" (CD_3OD).
